# Supplementary material for: Altered microRNA Transcriptome in Cultured Human Liver Cells upon Infection with Ebola Virus
Source: Int J Mol Sci. 2021 Apr 6;22(7):3792. doi: 10.3390/ijms22073792 (PMC8038836; doi:10.3390/ijms22073792)
Supplement: Supplementary file 1 [file ijms-22-03792-s001.zip › Supplementary_File/D_ KEGG_Analysis_Results/16-30nt_pathway_Makona-24h-Huh7_vs_Control-24h-Huh7_up.mature_mirna_targets/hsa_EnrichmentScore.pdf]

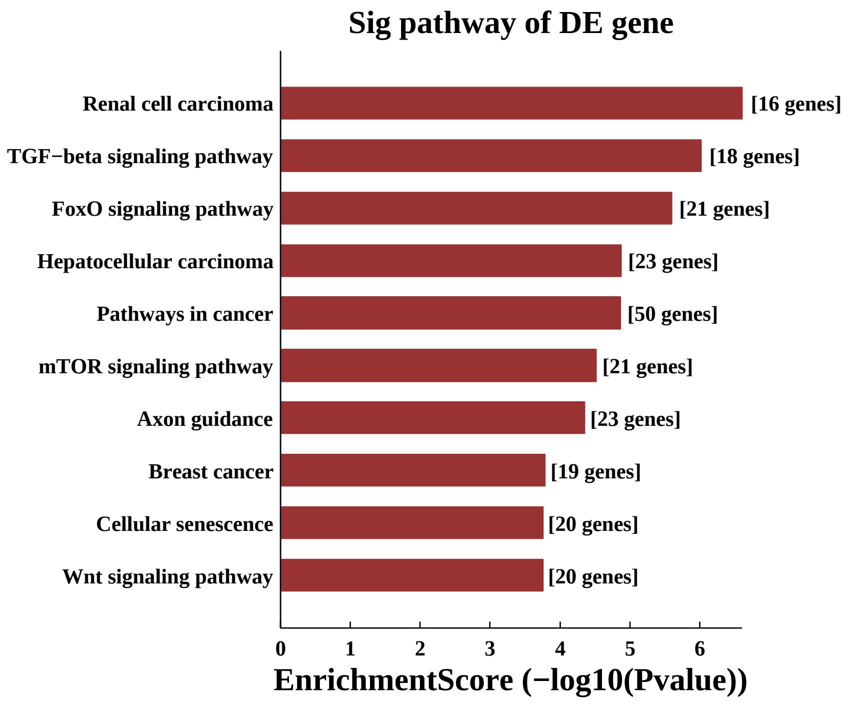
Sig pathway of DE gene
Renal cell carcinoma
TGF−beta signaling pathway
FoxO signaling pathway
Hepatocellular carcinoma
Pathways in cancer
mTOR signaling pathway
Axon guidance
Breast cancer
Cellular senescence
Wnt signaling pathway
[16 genes]
[18 genes]
[21 genes]
[23 genes]
[50 genes]
[21 genes]
[23 genes]
[19 genes]
[20 genes]
[20 genes]
0
1
2
3
4
5
6
EnrichmentScore (−log10(Pvalue))
